# Real-time PCR quantification of human complement C4A and C4B genes

**DOI:** 10.1186/1471-2156-7-1

**Published:** 2006-01-10

**Authors:** Agnes Szilagyi, Bernadett Blasko, Denes Szilassy, George Fust, Maria Sasvari-Szekely, Zsolt Ronai

**Affiliations:** 1 Institute of Medical Chemistry, Molecular Biology and Pathobiochemistry, Semmelweis University, Budapest, Hungary; 2 Department of Pharmacology and Pharmacotherapy, Semmelweis University, Budapest, Hungary; 3 3rd Department of Medicine, Research Lab, Szentágothai János Knowledge Centre, Semmelweis University, Budapest, Hungary; 4 Applera Hungary LTD, Budapest, Hungary

## Abstract

**Background:**

The fourth component of human complement (C4), an essential factor of the innate immunity, is represented as two isoforms (C4A and C4B) in the genome. Although these genes differ only in 5 nucleotides, the encoded C4A and C4B proteins are functionally different. Based on phenotypic determination, unbalanced production of C4A and C4B is associated with several diseases, such as systemic lupus erythematosus, type 1 diabetes, several autoimmune diseases, moreover with higher morbidity and mortality of myocardial infarction and increased susceptibility for bacterial infections. Despite of this major clinical relevance, only low throughput, time and labor intensive methods have been used so far for the quantification of C4A and C4B genes.

**Results:**

A novel quantitative real-time PCR (qPCR) technique was developed for rapid and accurate quantification of the C4A and C4B genes applying a duplex, TaqMan based methodology. The reliable, single-step analysis provides the determination of the copy number of the C4A and C4B genes applying a wide range of DNA template concentration (0.3–300 ng genomic DNA). The developed qPCR was applied to determine C4A and C4B gene dosages in a healthy Hungarian population (N = 118). The obtained data were compared to the results of an earlier study of the same population. Moreover a set of 33 samples were analyzed by two independent methods. No significant difference was observed between the gene dosages determined by the employed techniques demonstrating the reliability of the novel qPCR methodology. A Microsoft Excel worksheet and a DOS executable are also provided for simple and automated evaluation of the measured data.

**Conclusion:**

This report describes a novel real-time PCR method for single-step quantification of C4A and C4B genes. The developed technique could facilitate studies investigating disease association of different C4 isotypes.

## Background

**Figure 1:**
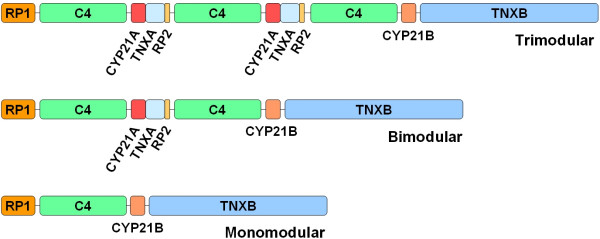
**Modular variations of human complement C4 and RP-C4-CYP21-TNX (RCCX) modules in the MHC class III region**. Each C4 (Complement C4) gene may code either the C4A or the C4B protein, and may consist of 21 kb (long, containing the HERV-K endogenous retrovirus) or 14.6 kb (short). RP: Ser/Thr protein kinase, CYP21: Steroid 21-hydroxylase, TNX: Extracellular matrix protein tenascin-X.

The complement system is a major constituent of innate immunity. Complement C4 plays an essential role in the activation cascades of the classical complement pathway as a subunit of the C3 and C5 convertases. C4 genes, located on the short arm of chromosome 6, are present either in a long (21 kilobasepair, kb) or in a short (14.6 kb) form, the long variant contains a 6.36 kb endogenous retrovirus HERV-K in its intron 9 [1,2]. These genes are deleted or duplicated together with the adjacent genes including RP (serine-threonine kinase), CYP21 (steroid 21-hydroxylase) and TNX (tenascin-X). The set of the four genes (RP, C4A or C4B, CYP21 and TNX) is referred to as the RCCX module [3]. The variation of the number of RCCX modules and sizes of the C4 genes leads to different RCCX length forms (Fig (1)): besides the monomodular L (long) and S (short), the bimodular (LL, LS, SS) and trimodular (LLL, LSS, LLS, LSL) types, the quadrimodular version (LLLL) was also described with a very low frequency. These length variants create more than 20 different haplotype combinations.

In addition to length variations, C4 genes have two main isotypes, C4A and C4B encoding functionally different proteins, as C4A is more reactive with targets containing free amino groups while C4B has a higher affinity to hydroxyl groups [4,5]. Most individuals have the same number of the two different C4 genes, while about 30% of the population has a lower level of either C4A or C4B proteins. The unbalanced production of C4A and C4B proteins has been associated to several diseases. Complete deficiency of the C4A or C4B gene in a haplotype module is referred to as C4A*Q0 and C4B*Q0, respectively. C4A*Q0, which is an essential constituent of the 8.1. ancestral haplotype, was found to be associated with systemic lupus erythematosus [6,7], insulin-dependent diabetes mellitus [8,9], myasthenia gravis [10], other autoimmune diseases and abnormalities of the immune system (reviewed in [11]). On the other hand, carriers of the C4B*Q0 have a highly increased risk for myocardial infarction [12], stroke [13] and an increased vulnerability for microbial infections [14]. Interestingly autism [14] and narcolepsy [15] have also been described to be associated with C4B deficiency although no responsible haplotype was identified.

For several decades the number of the C4A and C4B genes has been evaluated by phenotyping, i.e. by measuring the relative amount of the C4A and C4B proteins employing immunofixation electrophoresis. Direct quantification of C4A and C4B is more difficult as these genes are highly homologous with only five isotypic nucleotide differences [16,17]. This sequence variation can be detected by restriction fragment length polymorphism (RFLP) combined with Southern blot analysis [18]. Determination of the RCCX module number is possible with *Taq *I RFLP, while *Psh*A I RFLP was earlier used to define the C4A/C4B ratio [19]. Beside these techniques, there are several methods to demonstrate of the complete absence of C4A and C4B isoforms. C4 null alleles with non-expressed or absent C4A/C4B genes can be detected by high voltage agarose gel electrophoresis of carboxypeptidase and neuraminidase treated serum or plasma samples [20], as well as by RFLP analysis [21]. A rapid screening method was developed to determine the main form of the C4A null allele (C4A deletion) by long PCR [22]. Man and co-workers described a polymerase chain reaction (PCR) procedure with sequence specific primers (PCR-SSP) to determine the frequency of C4A and C4B null alleles in SLE patients [23].

Real-time PCR is one of the most applicable and up-to-date methods for DNA quantification, which allows to track the accumulation of the PCR product during the reaction. It provides the possibility to report the results as a threshold cycle (*C*_T_) value, which is the cycle number where the measured fluorescence reaches a given threshold. This threshold is adjusted to the initial section of the exponential phase of the amplification, thus the *C*_T _value is highly proportional to the copy number of the template DNA. Double-stranded DNA binding non-specific dyes, such as SYBR Green or sequence-specific probes (double-dye oligonucleotides) can be used to detect the amplified PCR products during the reaction. Employment of TaqMan probes is one of the most general applications of the latter approach, where fluorescence is generated based on the 5' nuclease activity of the *Taq *polymerase. The 3' quencher dye of the intact probe absorbs the light emitted by the reporter dye at the 5' end of the oligonucleotide and emits at much longer wavelengths that is not detected by the real-time PCR machine. On the other hand, the DNA polymerase cleaves the TaqMan probe during the extension step of the PCR, thus the emitted light of the reported dye is not quenched any more [24]. One of the most commonly used quencher dyes is TAMRA, an emerging alternative is however the employment of the MGB (minor groove binder) probes instead, which possesses several advantages. Although TAMRA emits at 582 nm, it is not completely dark at those wavelengths that are detected by the real-time PCR instruments. MGB is a non-fluorescent "dark" quencher, moreover it stabilizes the probe-template DNA duplex providing enhanced mismatch discrimination and higher precision at quantitative assays [25,26].

Here, we report a novel and rapid qPCR method for the gene dosage determination of the complement C4A and C4B genes. Our system employs real time PCR, and affiliates the two major applications of TaqMan probes: quantitative assay and SNP detection.

## Results

### Determination of the number of C4A and C4B genes

A novel robust and high throughput method was developed for C4 gene dosage determination by quantitative real time polymerase chain reaction (qPCR). Sequence specific TaqMan^® ^probes with minor groove binding (MGB) non-fluorescent quencher were applied to determine the number of the two isotypes, the quantitative assay of the RNase P gene was used as a reference. The copy number of C4A and C4B genes was determined in two separate tubes. Reaction mixture I contained the VIC-labeled C4A-specific probe and the FAM-labeled RNase P system, while the FAM-labeled C4B-specific probe and the VIC-labeled RNase P reference were applied in reaction mixture II (for sequences see Methods). To obtain the most consistent *C*_T _values, automatic baseline and manually adjusted threshold to the lowest possible level (approximately to 0.04 fluorescence (Δ*R*_n_) value) were applied. The number of C4A and C4B genes (*n*_C4A_, *n*_C4B_) was calculated according to equations (1) and (2),

nC4A=2CT(RF)−CT(C4A)+1qC4A:RF     (1)
 MathType@MTEF@5@5@+=feaafiart1ev1aaatCvAUfKttLearuWrP9MDH5MBPbIqV92AaeXatLxBI9gBaebbnrfifHhDYfgasaacH8akY=wiFfYdH8Gipec8Eeeu0xXdbba9frFj0=OqFfea0dXdd9vqai=hGuQ8kuc9pgc9s8qqaq=dirpe0xb9q8qiLsFr0=vr0=vr0dc8meaabaqaciaacaGaaeqabaqabeGadaaakeaacqWGUbGBdaWgaaWcbaGaee4qamKaeGinaqJaeeyqaeeabeaakiabg2da9maalaaabaGaeGOmaiZaaWbaaSqabeaacqWGdbWqdaWgaaadbaGaemivaqLaeiikaGIaeeOuaiLaeeOrayKaeiykaKcabeaaliabgkHiTiabdoeadnaaBaaameaacqWGubavcqGGOaakcqqGdbWqcqaI0aancqqGbbqqcqGGPaqkaeqaaSGaey4kaSIaeGymaedaaaGcbaGaemyCae3aaSbaaSqaaiabboeadjabisda0iabbgeabjabcQda6Gqaaiab=jfasjab=zeagbqabaaaaOGaaCzcaiaaxMaadaqadaqaaiabigdaXaGaayjkaiaawMcaaaaa@4F98@

nC4B=2CT(RV)−CT(C4B)+1qC4B:RV     (2)
 MathType@MTEF@5@5@+=feaafiart1ev1aaatCvAUfKttLearuWrP9MDH5MBPbIqV92AaeXatLxBI9gBaebbnrfifHhDYfgasaacH8akY=wiFfYdH8Gipec8Eeeu0xXdbba9frFj0=OqFfea0dXdd9vqai=hGuQ8kuc9pgc9s8qqaq=dirpe0xb9q8qiLsFr0=vr0=vr0dc8meaabaqaciaacaGaaeqabaqabeGadaaakeaacqWGUbGBdaWgaaWcbaGaee4qamKaeGinaqJaeeOqaieabeaakiabg2da9maalaaabaGaeGOmaiZaaWbaaSqabeaacqWGdbWqdaWgaaadbaGaemivaqLaeiikaGIaeeOuaiLaeeOvayLaeiykaKcabeaaliabgkHiTiabdoeadnaaBaaameaacqWGubavcqGGOaakcqqGdbWqcqaI0aancqqGcbGqcqGGPaqkaeqaaSGaey4kaSIaeGymaedaaaGcbaGaemyCae3aaSbaaSqaaiabboeadjabisda0iabbkeacjabcQda6iabbkfasjabbAfawbqabaaaaOGaaCzcaiaaxMaadaqadaqaaiabikdaYaGaayjkaiaawMcaaaaa@4FDB@

The real number of C4A and C4B genes is the rounded value of *n*_C4A _and *n*_C4B _respectively, *C*_*T*(RF)_, *C*_*T*(C4A)_,*C*_*T*(RV) _and *C*_*T*(C4B) _are the determined threshold cycle values of the FAM labeled reference (RNase P), the C4A, the VIC labeled reference and the C4B reactions. *q*_C4A:RF _and *q*_C4B:RV _are the efficiency quotients for VIC labeled C4A and FAM labeled reference in reaction I, and for FAM labeled C4B and VIC labeled reference in reaction II, respectively.

### Calculation of efficiency quotients

In principle two copies of the C4A or C4B genes should result in the same signal as that measured for the RNase P gene, as this latter one is known to be present in a single copy in both members of the homologous chromosome pairs. In practice, however, the efficiency of the primers used for the C4 and RNase P genes is not equal, moreover additional variations are caused by the sequence differences of allele specific probes and by the application of different dyes. Therefore, the efficiency quotients should have been determined experimentally, which was a crucial part of our study, as the subsequent accurate genotype determination is based on these data. This optimization was carried out by the analysis of 65 samples in 7 separate reactions. Applying the measured *C*_T _values the efficiency quotients (*q*) could be determined based on three assumptions: (1) too high *q *values could be excluded by the simple fact that the number of the C4A or C4B gene cannot be zero if significant fluorescence could be detected in the appropriate reaction. (2) On the other hand, the use of too low *q *values could also be avoided because the sum of the number of C4A and C4B genes had been described to be practically never higher than 6 in a Caucasian population [27]. (3) The number of the C4A and C4B genes can be only an integer. Applying equations (1) and (2) in combination with the above assumptions number of C4A and C4B genes of the 65 samples as well as the accurate values of the efficiency quotients (*q*) have been determined. The obtained efficiency values showed some intra- and inter-assay fluctuation, which was probably the consequence of pipetting inaccuracy (see Table 1). The standard deviation values depict the intra-assay error, while the inter-assay inaccuracy is demonstrated in the last row of the Table 1. To assess this error rate we pooled the data of the 7 experiments and calculated overall efficiency ratios for the total number of 65 samples. Based on these results and the analyses carried out for the investigation of our Hungarian population (*N *= 118 samples, 11 more assays), the efficiency quotient of the C4A (VIC) and the RNase P (FAM) reactions was found to be *q*_C4A:RF _= 0.39 ± 0.04, while that of the C4B (FAM)-RNase P (VIC) reactions was *q*_C4B:RV _= 0.75 ± 0.11 under applied conditions.

**Table 1:** "Individual" (rows 1–7) and "overall" (last row) efficiency quotients (*q *values) and the corresponding error of C4A and C4B gene qunatification

PCR	*q*_C4A:RF_	*q*_C4B:RV_	Average error
1	0.38 ± 0.03	0.64 ± 0.04	0.13
2	0.40 ± 0.02	0.71 ± 0.03	0.07
3	0.36 ± 0.04	0.67 ± 0.07	0.15
4	0.39 ± 0.03	0.70 ± 0.03	0.10
5	0.36 ± 0.02	0.66 ± 0.03	0.06
6	0.41 ± 0.03	0.67 ± 0.05	0.09
7	0.42 ± 0.02	0.65 ± 0.05	0.09

Overall	0.39 ± 0.04	0.67 ± 0.05	0.13

### Reliability of C4A and C4B gene quantification

To analyze the reliability and reproducibility of the developed qPCR system, we investigated the error of the C4A and C4B gene quantification by calculating either with the overall *q *of our 7 pilot experiments or with the values defined by each individual experiment ("individual *q*") respectively. Error of the gene number quantification was calculated as the absolute value of the difference of the exact (equations (1) and (2)) and rounded *n *values according to equation (3).

**Figure 2:**
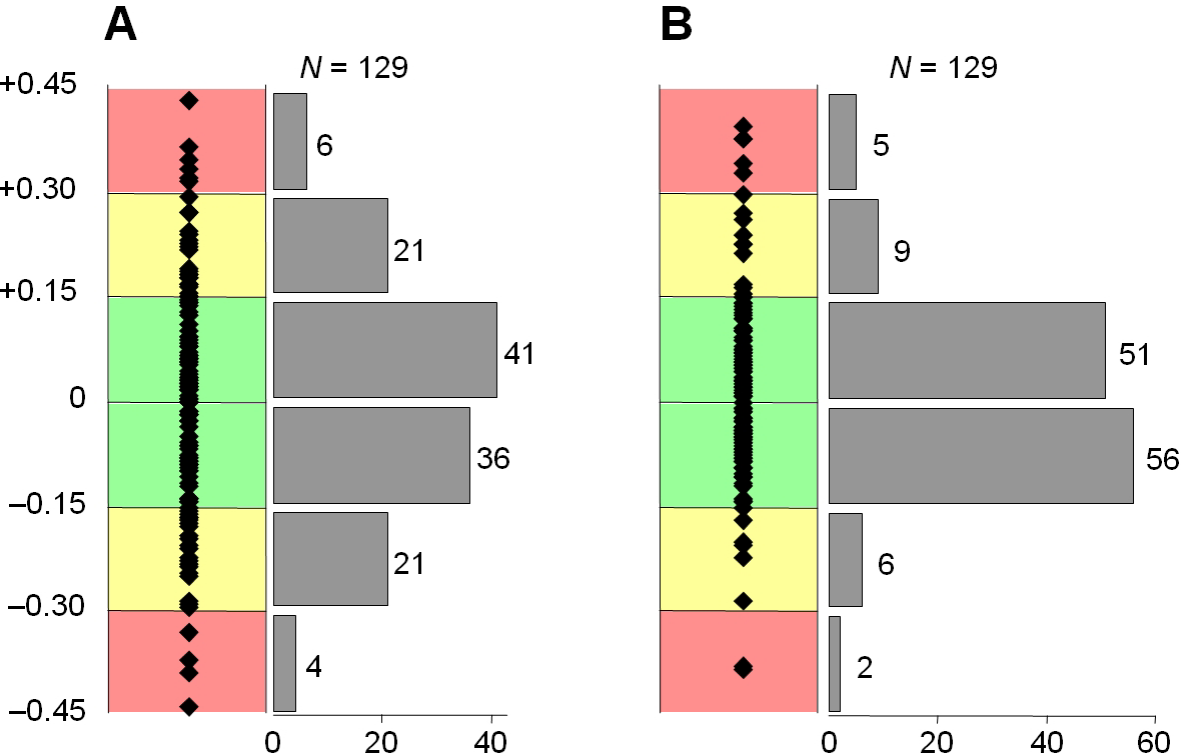
**Precision of the determination of the C4A and C4B gene number. Panel A: calculation by the "overall *q*" values, Panel B: calculation by the "individual *q*" values**. The position of the squares indicate the distance of the calculated gene number from an integer (i.e. the error of the analysis). Green area: result is of high reliability, yellow area: acceptable result, red area: doubtful result. The gray bars show the number of analyzed alleles corresponding to the clusters of different reliability.

*e = *|*n*_C4 _- int(*n*_C4 _+ 0.5)|     (3)

where *e *is the error, *n*_C4 _is the calculated value of *n*_C4A _or *n*_C4B_, and int(*n*_C4 _+ 0.5) is the rounded integer. The last column of Table 1 shows the average error of the gene dosage determination: rows 1–7 demonstrate the individual values for each experiment using the "individual *q*-s", while the average error obtained by using the overall efficiency quotients is shown in the last row. The difference between the two approaches is highlighted in Fig (2). The total number of C4 gene quantifications in the pilot assays was 129 as one of the 65 persons possessed no C4A gene. Panel A of Fig (2) shows that applying the overall *q *values for all the 129 reactions, 60% of them were in the absolutely reliable range of *e *< 0.15, and only 8% of the results had an *e *≥ 0.3 value. If the "individual *q *values" of each reaction were used, the percentage of the absolutely reliable results was 83%, and the ratio of reactions with *e *≥ 0.3 was 5% (Panel B of Fig (2)). As expected, the average error is higher using the global *q *values. This difference was however small enough to provide an undoubted gene number determination as application of either the "overall" or the "individual" *q *values gave the same C4A and C4B gene numbers for all the 65 samples. However it is suggested to determine to "individual" *q *values for each experiment by MHC.EXE and/or MHC.XLS (see below) to achieve the highest reliability of C4 quantification.

### Reliability of gene number determination in a large DNA concentration range

**Figure 3:**
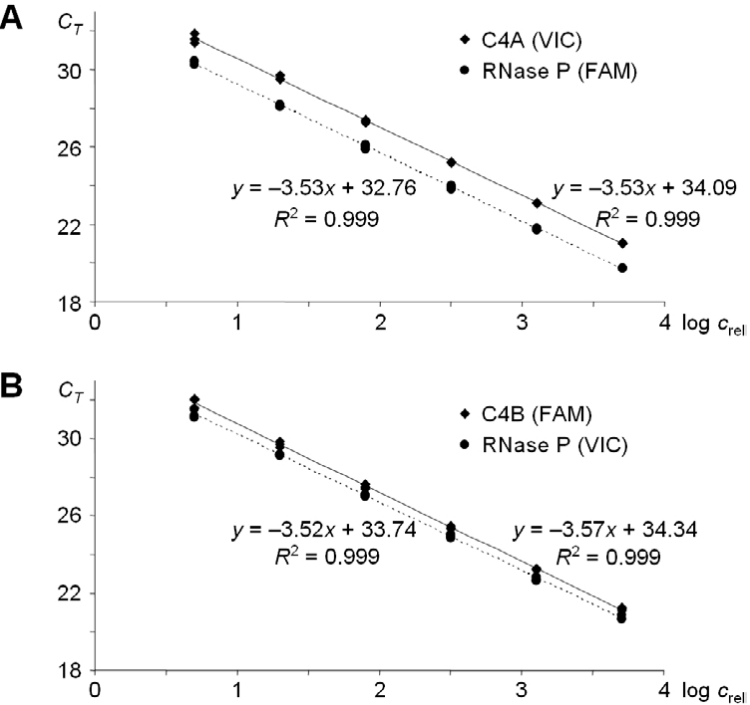
**Standard curves for the C4A and C4B gene number quantification**. Panel A: Standard curve of "Reaction Mixture I.": VIC labeled C4A specific and FAM labeled RNase P specific probes, Panel B: Standard curve of "Reaction Mixture II.": FAM labeled C4B specific and VIC labeled RNase P specific probes. Linear regression analysis equation and coefficient of correlation are shown.

The experiment presented in Fig (3) proves that the developed qPCR is reliable practically independently of the amount of DNA template. A quartering series of dilutions was prepared covering the range of 0.3–300 ng genomic DNA of a sample possessing 2 C4A and 2 C4B isotypes. Three parallel measurements were carried out at each concentration. Figure 3A shows the *C*_*T *_values of the C4A (VIC) and the RNase P (FAM) (reaction mixture I), 3B demonstrates those of the C4B (FAM) and the RNase P (VIC) reactions (reaction mixture II) as the function of the logarithm of the relative DNA template concentration. It can be observed that the fitted lines are practically parallel (i.e. slope values are almost identical), moreover the *R*^2 ^= 0.999 values demonstrate that the data points fit very accurately to the lines. Based on this, any dilutions of DNA templates resulting in *C*_*T *_values between 20 and 30 seem to be applicable in this genotyping system.

### Application of the developed qPCR system for C4 gene dosage analysis in a Hungarian population

The number of C4A and C4B genes was determined by using the elaborated qPCR in a healthy Hungarian population (*N *= 118). The obtained data (Table 2, "present work") were compared to the results of an earlier study [28] (Table 2 "published data"), which employed the Southern-blot based method of Blanchong et al [19]. Statistical analysis of data obtained by the novel qPCR and by the previously applied Southern-blot analysis did not show significant difference. In certain categories however some discrepancy could be observed. Therefore, a set of 33 samples was analyzed by the described qPCR method, as well as by a novel independent, capillary electrophoresis based technology (Szilagyi et al., submitted 2005), and a one-to-one comparison of the obtained gene dosages was carried out. After repetition of the analysis of three samples with doubtful results, a perfect consistency of the two methods was observed proving the accuracy of the novel real-time PCR based methodology.

**Table 2:** C4A and C4B gene dosage in a healthy cohort determined by the presented qPCR method. Comparison to earlier data obtained by Southern-blot analysis [28]

C4A genes			C4B genes		
Gene dosage	Present work	Published data	Gene dosage	Present work	Published data

	Number (%)			Number (%)	

0	2 (1.7)	2 (1.6)	0	1 (0.8)	1 (0.8)
1	18 (15.3)	26 (20.3)	1	24 (20.3)	26 (20.3)
2	65 (55.1)	73 (57.0)	2	75 (63.6)	90 (70.3)
3	30 (25.4)	24 (18.8)	3	18 (15.3)	10 (7.8)
4	3 (2.5)	3 (2.3)	4	0 (0.0)	1 (0.8)

Total	118 (100)	128 (100)		118 (100)	128 (100)

χ^2 ^test	*p *= 0.7023			*p *= 0.3632	

### Software provided for calculations

An Excel sheet (MHC.XLS) and a stand-alone software (MHC.EXE) were designed to improve the processing of the measured *C*_*T *_values. The software reads the "csv" file created by the "Export Ct values..." function of the Sequence Detection Software (SDS) of the Real Time PCR Instrument of Applied Biosystems. It optimizes the *q *values in a default range of 0.19–1.59 for both *q*_C4A:RF _and *q*_C4B:RV_, however these ranges can be modified by the user. The measure of stringency ("error level") has to be entered, and the software creates a data file containing the C4A and C4B gene numbers and the reliability of the results. "+++" means that *e *(see equation 4) is smaller than the error level, "++" shows that *e *is higher than this limit, but lower than its double. One or zero "+" shows even higher distance of the calculated number from an integer, in this case the gene dosages are not determined. The software can export sample names and *C*_*T *_values in the format required by the Excel sheet.

The Excel file, MHC.XLS calculates the same results in a more user-friendly Windows based environment. In this case the user has to type the *q *values, however the calculated optimal ratios are shown. Moreover red background of these cells warns if the entered numbers are too far away from the optimal values. In this file green, yellow and red dots show the reliability of the calculations of the number of the C4A and C4B genes, based on the entered "error limit" and the same assumptions described above. Further details about the software and the Excel sheet can be read in their manual that can be downloaded together with the files as "additional files of the paper".

## Discussion

Real-time PCR is a useful tool for quantitative measurements, thus it is readily applicable for gene expression analyses as well as for the investigation of gene dosage (i.e. for the determination of the copy number of different genes in the genome or in transgenic organisms). Melo et al. developed a real-time PCR based system for the quantification of glucocorticoid receptor alpha isoform. Similarly to our observation they demonstrated that the method is reliable in a very wide template concentration range of higher than 3 orders of magnitude [29]. Bubner and Baldwin reviewed the use of real-time PCR for determining copy number and zygosity in transgenic plants. It was shown that carefully optimized reaction conditions and the application of MGB probes in combination with the comparative method (2−ΔΔCT
 MathType@MTEF@5@5@+=feaafiart1ev1aaatCvAUfKttLearuWrP9MDH5MBPbIqV92AaeXatLxBI9gBaebbnrfifHhDYfgasaacH8akY=wiFfYdH8Gipec8Eeeu0xXdbba9frFj0=OqFfea0dXdd9vqai=hGuQ8kuc9pgc9s8qqaq=dirpe0xb9q8qiLsFr0=vr0=vr0dc8meaabaqaciaacaGaaeqabaqabeGadaaakeaacqaIYaGmdaahaaWcbeqaaiabgkHiTiabgs5aejabgs5aejabdoeadnaaBaaameaacqqGubavaeqaaaaaaaa@33F1@) provided the possibility to detect as low as two-fold differences which is a key issue in gene dosage analyses [25].

**Figure 4:**
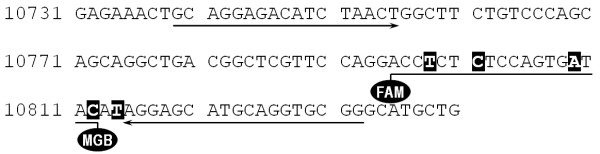
**Position of TaqMan probe and primers for C4 gene number analysis**. A short part of the C4B gene is shown [GenBank:U24578]. Arrows: position of the primers, underlined sequence with FAM at 5' end and MGB at 3' end: position of the TaqMan probe. White letters in black boxes: 5 single nucleotide polymorphisms distinguishing the C4A and C4B genes.

There are two methods to monitor the amount of the PCR-products during the reaction: the application of intercalator dyes (e.g. SYBR Green) demonstrate the presence of the double stranded DNA, thus it can be used in any system. The design and employment of sequence specific probes (e.g. TaqMan) is the other approach. Besides the accurate and specific quantification of the PCR product of interest this method is readily applicable to detect as small as a single base change in the DNA sequence thus it can be used for SNP genotyping [24,30]. These two ideas were combined in our system developed for the determination of the number of C4A and C4B genes. There are only 5 isotypic base changes in a 17-bp-long sequence of the two genes differentiating them from each other (see Fig (4)). C4A and C4B specific TaqMan probes were designed covering four of these SNPs, and the two probes were labeled with different dyes (VIC and FAM respectively). Although we optimized the presented qPCR method as a two-tube-system for the accurate quantification of the C4 genes, the purpose of labeling the two TaqMan probes with different dyes was to provide a further possible application for large-scale screening. It is a single-tube-approach, where the C4 specific probes are used in the same reaction and no RNase P reference is applied. Although in this case only the ratio of the number of C4A and C4B genes can be determined, the system is of double throughput, as only three (and not six) parallel measurements are necessitated. This simplified technique is suitable for preliminary screening as well as for the determination of the lack of the C4A or C4B isotype, whereas the double-tube system described in detail is to be used for the determination of the definite number of the C4 genes.

The quantification of C4A and C4B genes is of great clinical as well as theoretical importance, because either the deficiency or the exceeding amount of any of the two C4 variants may adversely influence immune processes. Although the sequence of the two isoforms differs in less than 1%, this variance alter their hemolytic and serological reactivity as well as their affinity to antigens and immune complexes [27]. The disease association of the C4A*Q0 or C4B*Q0 phenotypes have been widely investigated. C4A*Q0, an essential constituent of the 8.1 ancestral haplotype [11] was found to be related to systemic lupus erythematosus [31-34], Graves' disease [35] and systemic sclerosis [36]. In contrast the association of the C4B*Q0 and shorter life-expectancy [37], increased susceptibility for myocardial infarction [12], stroke [13], autism [38], Henoch-Schonlein purpura glomerulonephritis [39], bacterial meningitis[40], angio-oedema and "lupus-like" disease [41], and bacteremia with encapsulated organisms [42] and meningococcal disease [43] was also described.

This wide range of diseases underlines the relevance of a simple and high throughput method for C4 gene dosage analysis to improve our knowledge about the role of functionally different isotypes in physological as well as in the above mentioned pathological immune procedures. RFLP and gel electrophoresis based methods developed earlier [18,20] are of low throughput, moreover they are technically difficult and labor intensive. RFLP analysis in combination with Southern-blot is the only method at present which is suitable for characterization of the whole RCCX module, but it requires a large amount of DNA and the employment of radioisotopes.

Our report presents a rapid qPCR technique for the determination of the number of C4A and C4B genes. The developed method is a single step procedure, where no subsequent post-PCR analyses are required. Computational applications are also provided for automated allele-calling procedure, which makes the evaluation of the results fast, simple and reliable. The determined C4 gene dosages were compared to the data of an earlier study that investigated the same group of healthy Hungarian subjects applying Southern blot for C4A and C4B gene quantification. Moreover 33 samples were analyzed by two independent methods. There was no significant difference between the results of these studies underlining the accuracy of our novel method. Although the demonstrated technique has some limitations, for example it is not suitable for the identification of the non-functional C4 genes caused by 1- or 2-bp-deletions [44], this comprehensive system could facilitate the investigation of complement C4A and C4B genes in the future.

## Methods

Genomic DNA was isolated from peripheral blood using the Flexigene DNA isolation kit (Qiagen). Primers and fluorogenic probes for the 5' nuclease assay were designed by the Primer Express software. Fig (4) shows a short segment of the sequence of complement C4B gene [GenBank:U24578] demonstrating the position of the primers (arrows) and the TaqMan probe. The black boxes indicate those nucleotides that are different in the two isoforms, this sequence variation was used to design the C4A and C4B specific probes. For quantifying the C4A and C4B genes two separate reactions were used, three parallels were carried out for each measurement. Both reaction mixtures contained 6 μM forward (5' GCA GGA GAC ATC TAA CTG GCT TCT 3') and 6 μM reverse (5' CCG CAC CTG CAT GCT CCT 3') primer (see Fig (4)), 1× TaqMan Universal PCR Master Mix (AmpliTaq Gold^® ^DNA Polymerase, dNTPs with dUTP, Passive Reference, No AmpErase UNG^®^) and genomic DNA template. Reaction mixture I contained furthermore the C4A specific TaqMan probe (5' VIC-ACC **C**CT **G**TC CAG TG**T** TA**G**-MGB 3'; MGB: minor groove binding non-fluorescent quencher) and the FAM-labeled 1× RNase P Detection Mix (ABI Cat. No. 4316831), while the FAM-labeled C4B specific TaqMan probe (5' FAM-ACC **T**CT **C**TC CAG TG**A** TA**C**-MGB 3') and the VIC-labeled 1× RNase P Detection Mix (ABI Cat. No. 4316844) was added to reaction mixture II in a total volume of 25 μl. (The bold and underlined letters show the sequence differences of the two probes corresponding to the nucleic acid variations that distinguish the C4A and C4B genes.) DNA amplification was carried out in an ABI 7500 Real Time PCR System. Thermocycle was initiated by incubating the mixtures at 95°C for 10 minutes to denature genomic DNA and to activate AmpliTaq Gold^® ^DNA Polymerase. This was followed by 40 cycles of two steps of 95°C for 15 sec and 60°C for 1 minute, the fluorescence intensity was measured during the step of 60°C.

DNA samples of 173 healthy Hungarian individuals were used for the present study. These individuals participated in a regular medical survey and gave their informed consent for the use of their sample for the study. For ethical reasons after their computer registration the data were unlinked from the subjects so their identities could not be traced. The study was approved by the Ethical Committee of the Semmelweis University (Budapest, Hungary)

## Authors' contributions

AS and BB carried out the major part of the experimental work and helped to draft the manuscript. DS designed the C4 gene specific primers and TaqMan probes. GF conceived of the study, MS and GF participated in the design and coordination of the study, helped to evaluate the results and to draft the manuscript. ZR took part in the experimental work and in the manuscript preparation, designed the Excel sheet and the software for data analysis. All authors read and approved the final manuscript.

## Supplementary Material

Additional File 1 Sample data file as exported by the software of the ABI real-time PCR instrument. Click here for file

Additional File 2 User's manual for mhc.xls and mhc.exe Click here for file

Additional File 3 This program can be used to calculate the number of the C4A and C4B genes. The input file has to contains the *C*_T _values exported by the software of the ABI real-time PCR instrument. The program exports the data in the format that can be copied into the Excel sheet. See details in mhcmanual.doc Click here for file

Additional File 4 This Excel file can be used to calculate the number of the C4A and C4B genes based on the measured *C*_T _values. See details in mhcmanual.doc Click here for file
