# Supplementary material for: Real-time PCR quantification of human complement C4A and C4B genes
Source: BMC Genet. 2006 Jan 10;7:1. doi: 10.1186/1471-2156-7-1 (PMC1360677; doi:10.1186/1471-2156-7-1)
Supplement: Additional File 2 — User's manual for mhc.xls and mhc.exe [file 1471-2156-7-1-S2.doc]

# User’s manual

## MHC.XLS

MHC.XLS is a Microsoft Excel sheet designed for automatic determination of the number of C4A and C4B genes based on the measured *C*_T_ values.

*Data input*

The first two columns (A, B) contain information about the experiment and the DNA sample for documentation purposes.

The appropriate three parallel measured ***C*_T_ values** for the four TaqMan systems **have to be put into C4A (VIC), RNase P (FAM), C4B (FAM) and RNase P (VIC) columns respectively**. You can either type the values into the Excel-sheet or if you use an ABI Real-time PCR System with Sequence Detection Software (Applied Biosystems) and our MHC.EXE executable, then you can convert data into the appropriate format and simply paste the *C*_T_ values here.

Besides these, the **efficiency quotients (*q*) and the error level have to be entered** to begin data analysis. We suggest to start with 0.39 for C4A (VIC) / RNase P (FAM), 0.76 for C4B (FAM) / RNase P (VIC) and 2.00 for RNase P (FAM) / RNase P (VIC) or by using the values determined by MHC.EXE, later however several signals will help to find the optimal values corresponding to your experiment (see below).

**Error level is recommended to set to 0.15**, as this value provides acceptable reliability, however based on user’s expectations any value between 0.1 and 0.2 can be rational.

*Optimization of the Efficiency quotients*

The “calculated efficiency quotients” show the *q* values determined based on the data you entered. It is suggested to **correct your values according to these numbers**, as any slightly different experimental conditions as well as pipetting inaccuracies can alter relative efficiencies.

If the entered values were very far from the optimal range, the background of these **cells turn**s **into red**. It happens, if (1) the number of C4 genes is calculated to be 0 although fluorescence was detected in case of any sample; (2) the total number of C4 genes is higher than 8; (3) the efficiency quotient of the C4A and C4B specific reactions calculated by other *q* values and that determined by the measured data are considerably different.

It’s important to note that the “calculated efficiency quotients” are determined based on the reliable reactions only (samples with green or yellow signs and acceptable standard deviation of the parallel values – see below), thus any individual error of a single reaction doesn’t disturb the calculation of the whole experiment.

*Results and messages*

If the C4A and C4B genes can be reliably quantified, their copy numbers appear in the “Genotye C4A and C4B” columns.

The **control columns** depict the **reliability of the reaction showing a green, yellow or red dot**. Green dots appear in Columns “C4A” and “C4B” if the calculated copy number is in the range of an integer ± the error level. The color of the dots is yellow, if the obtained value is outside of this range but the discrepancy is not higher than the double of the error level. Red dots indicate even higher error. The RNase P and C4 columns show the difference between the expected and measured efficiency quotient values based on the same logic described above. **If any of the four dots is red, it shows that the reliability of the reaction is not acceptable, thus the calculated number of C4 genes is not shown.** The strictness of the calculation is however determined by the user by setting the error level.

An other important sign of a reliable reaction is the small standard deviation of the parallel values. It is also taken into consideration: **if the standard deviation of the three parallel *C*_T_ values is high**er than the double of the error level, then **the background of the appropriate cells turn into red and the gene number is not shown**. However the *C*_T_ value that doesn’t fit can be omitted, as missing values do not disturb the calculation.

According to our experiments too small DNA template amounts do not allow a reliable C4 quantification. Consequently *C*_T_ values higher than 32 are indicated by yellow background, although – if all other conditions are fulfilled – gene number is calculated.

The **“Export” worksheet** can be used to print the results or to copy the gene numbers to your data base. In this work sheet you will find the name of the samples and the determined gene dosage in a simple format that can be used to transport the results to any software applied for downstream data processing.

We suggest using a novel mhc.xls file for each experiment, however the estimation of the optimal *q* values will only be robust enough if it is based on at least 7–8 samples. Consequently if you analyze fewer samples in a single experiment, then you should evaluate the obtained data in a single file, though due to inter-assay inconsistencies the reliability of the results will be slightly lower (see Fig (**1**) of our paper).

## MHC.EXE

MHC.EXE is a DOS executable that can be used to automatically calculate the number of the C4A and C4B genes based on the *C*_T_ values exported by the Sequence Detection Software (Applied Biosystems), moreover this software converts the data into a text file that can be simply pasted into MHC.XLS.

*Data input*

The software reads the “.csv” file created by the “File / Export / *C*_T_” function of the Sequence Detection Software (Applied Biosystems) (SDS).

*First use*

When you use MHC.EXE for the first time, you are prompted to enter the **name of the 4 detectors** used in the SDS software for the C4A and C4B specific, and those for the FAM and VIC labeled RNase P system, respectively. Names are not case sensitive.

Moreover you will be asked to define the **decimal separator** used in your country (“,” or “.”) as it will be taken into consideration when data are exported. Please note however, that regardless of this selection in MHC.EXE you always have to use “.” when entering a decimal fraction.

The name of the detectors and the decimal separator is stored in MHC.INI created at this time. Consequently if you need to modify these data (e.g. you modified the name of the detectors in the SDS software) then you can do this by editing MHC.INI by any text editor. Alternatively you can simply delete this file, and at the next use of MHC.EXE you will be asked again to enter the name of the detectors.

*Data processing*

First, you will be asked to **enter the name of your data file**. Please note, that MHC.EXE is a DOS based software allowing the application of 8-character-long DOS file-names only. (If the name of the “.csv” file is longer than 8 characters than you have to enter the first 6 characters followed by “~1” and the extension of the file. E.g. instead of “experiment_234.csv” you have to type “experi~1.csv”

Then you have to enter the **error level**. (See explanation in the description of MHC.XLS.)

After this the software reads the data and checks the standard deviation of the parallel values. If the StdDev is higher than the double of the error level then you **can delete one of the three parallels that doesn’t fit**. Values that you erase here won’t be used for the calculation and won’t even be written into the exported data file.

As a next step you are asked if you want to modify the **range where the optimal efficiency quotients will be searched for** (“Enter” means no, as according to our experiences it is not required). Default range for C4A (VIC)–RNase P (FAM) efficiency quotient is 0.19–0.59, that for C4B (FAM)–RNase P (VIC) is 0.56–0.96.

After this the software tries to find the best quotient values and calculates the number of C4A and C4B genes. You will be asked to enter the **name of a file that will contain the results**. This file contain similar information to that shown by MHC.XLS. “+++”, “++” and “+” is written instead of green, yellow and red dots respectively, and “too high StdDev!” message warns the user if the standard deviation of the three parallels is higher than the double of the error level. However MHC.EXE shows the result of the quantification even in this case.

As a final step you can convert the *C*_T_ values to the format of MHC.XLS. The resulted data file contains the list of the sample names in the upper part and the *C*_T_ values in the lower part, you have to copy and paste these data separately into MHC.XLS.

MHC.EXE and MHC.XLS are developing applications. If you experience any bugs or difficulties, please email to [ronai@puskin.sote.hu](mailto:ronai@puskin.sote.hu).

Although MHC.EXE and MHC.XLS are free to use, we ask you to register by sending an e-mail to [ronai@puskin.sote.hu](mailto:ronai@puskin.sote.hu). If you do so, we will send the appearing updated versions of the software and the Excel sheet to you.

When using MHC.EXE or MHC.XLS, please cite A. Szilagyi, B. Blasko, D. Szilassy, G. Fust, M. Sasvari-Szekely and Z. Ronai, **Real-time PCR quantification of human complement C4A and C4B genes**, *BMC Genetics*, 2005
